# Cardiac Fibroblast to Myofibroblast Phenotype Conversion—An Unexploited Therapeutic Target

**DOI:** 10.3390/jcdd6030028

**Published:** 2019-08-16

**Authors:** Michael P. Czubryt

**Affiliations:** Department of Physiology and Pathophysiology, Rady Faculty of Health Sciences, University of Manitoba and Institute of Cardiovascular Sciences, St Boniface Hospital Albrechtsen Research Centre, R4008 351 Tache Avenue, Winnipeg, MB R2H 2A6, Canada; mczubryt@sbrc.ca; Tel.: +204-235-3719; Fax: +204-233-6723

**Keywords:** fibroblast, myofibroblast, extracellular matrix, fibrosis, phenotype, heart failure, therapy

## Abstract

Fibrosis occurs when the synthesis of extracellular matrix outpaces its degradation, and over time can negatively impact tissue and organ function. In the case of cardiac fibrosis, contraction and relaxation of the heart can be impaired to the point of precipitating heart failure, while at the same time fibrosis can result in arrhythmias due to altered electrical properties of the myocardium. The critical event in the evolution of cardiac fibrosis is the phenotype conversion of cardiac fibroblasts to their overly-active counterparts, myofibroblasts: cells demarked by their expression of novel markers such as periostin, by their gain of contractile activity, and by their pronounced and prolonged increase in the production of extracellular matrix components such as collagens. The phenotype change is dramatic, and can be triggered by many stimuli, including mechanical force, inflammatory cytokines, and growth factors. This review will explore fibroblast to myofibroblast transition mechanisms and will consider the therapeutic potential of targeting this process as a means to arrest or even reverse cardiac fibrosis.

## 1. Introduction

Nearly every tissue in the body is supported by a complex extracellular matrix (ECM) that comprises, in part, the tissue stroma—that portion of the tissue that is not directly responsible for its function, but rather provides necessary structure and connectivity. The ECM is analogous to the walls, ceilings and floors that form a commercial building, and that provide a place for work to occur while not carrying out the work directly. Although the general structure of buildings may be similar, the specific materials that go into their construction vary according to the role of the building: laboratories, restaurants and banks bear a superficial resemblance, and are built according to common underlying engineering principles, but they also have significant variability in their specific make-up necessitated by the unique roles they fulfill. Like these buildings, the ECM of different tissues and organs may have clear similarities—the relative proportion of type I collagen is typically high across varied tissue types, for example—but also differences in their specific constituents such as fibrillar versus reticular collagen, their relative amounts, and their orientation. This variation likely reflects the need for the tissue to withstand, interpret and transmit variable physical forces, while at the same time providing different mechanical properties to different organs—contrast, for example, the relatively stiff myocardium to the relatively soft lung, which experiences widely different physical forces and tissue deformations.

Just as buildings must be repaired when damaged in order to maintain their integrity and function, the ECM is continually being remodeled to maintain its structure, to withstand physical impairment, and to adapt to changing environmental forces. In response to tissue injury, a well-defined healing process occurs involving the steps of inflammation, cell proliferation and remodeling [1,2]. When all goes well, healing is complete and scar-free. In contrast, impairment of the healing process can result in exaggerated remodeling leading to tissue fibrosis and organ dysfunction [3]. Fibroproliferative disorders have been implicated as contributing to 45% of human mortality [4]. This is perhaps not surprising, considering the central importance of ECM to tissue structure and function, however in many fields it is only relatively recently that fibrosis has taken prominence as a focus for therapeutic development [5]. Indeed, the development of anti-fibrotic treatments is arguably one of the hottest topics in medicine today, across organ systems. Yet, despite the significant impact of fibrosis on human health, at present there are only two anti-fibrotic medications on the market, with a narrow target population of patients with idiopathic pulmonary fibrosis; thus, the current fervent interest in anti-fibrotics is clearly justified [5]. This review examines the potential for attenuation of fibrosis by arresting the activation of fibroblasts, with a focus on cardiac fibrosis.

## 2. Extracellular Matrix and Fibrosis

The ECM is comprised of a complex assortment of collagens, proteoglycans, glycoproteins and matricellular proteins that together provide the structural support required by the functional cells of the tissue, while also providing mechanical characteristics appropriate to the tissue such as stiffness and deformability. Tissues such as the lung and liver are typically “soft” while others such as muscle or tendons may be much stiffer. The relative stiffness of a tissue in turn can impact on the function and stress response of both native cells and exogenous cells introduced for therapeutic purposes [3,6,7,8,9]. The overall structure of the ECM can also vary widely according to function—from highly organized fibrillar structures like tendons that serve to flexibly anchor muscle to bone, to the mesh-like reticular basement membrane of many tissue types, including the endothelium where the ECM supports a barrier function.

The ECM is synthesized and maintained by stromal cells embedded throughout the tissue or organ. Most often, these cells are fibroblasts, although there is significant ongoing debate regarding the nature of fibroblasts, their nomenclature and their heterogeneity across tissue types [2,10,11,12]. Other cell types may also contribute to ECM synthesis, such as hepatic stellate cells, vascular smooth muscle cells and fibrocytes, depending on the particular tissue in question [12]. A common theme that has emerged is that, while ECM and the stromal cells that generate it may be linked by a common underlying role, the highly variable mechanical requirements of different tissue types has resulted in significant heterogeneity in these stromal cells as well as in the specific ECM that they produce. In the heart, even atrial versus ventricular fibroblasts appear to have important differences in their biology and capacity to generate ECM [13,14].

The heart, which pumps over 100,000 times per day in humans, undergoes dramatic morphological changes with each contraction cycle. The underlying ECM must be robust enough to withstand this wear and tear, yet flexible enough so as not to interfere with cardiac contraction and relaxation. Decellularization of the heart, in which cells are removed by detergent perfusion, leaves behind an ECM structure that is remarkable in how much it resembles the intact heart, demonstrating the role played by the ECM in maintaining cardiac structure. Electron microscopy reveals that cardiomyocytes are in intimate contact with ECM fibers throughout the myocardium [15,16]. Our studies have found that the murine cardiac ECM accounts for approximately 25% of the mass of the heart [17]. The intact nature of the decellularized ECM has spurred attempts to remove ailing cells from failing hearts with the aim of recellularizing the heart with healthy donor cells in order to restore function [18,19].

It is important to note that the ECM is not static—it is constantly being broken down, due to the combined actions of physical damage and matrix metalloproteinases (MMPs), and replaced via *de novo* synthesis by stromal cells such as fibroblasts [20,21,22]. The half-life of collagen in macro structures in which collagen is a major and fundamental structural component, including articular cartilage and intervertebral discs, has been estimated from aspartate racemization rates as being on the order of 1–2 centuries [23,24]. In contrast, collagen half-life measured by an isotope labelling method in the muscle, skin and gut—tissues in which collagen plays a stromal/ECM role—ranged from 45 to 244 days, suggesting that it is being actively turned over in these tissues [25]. The ECM also plays more than simply a structural role—many growth factors are bound to the ECM, such as Transforming Growth Factor β (TGFβ), which is sequestered in a latent form that can be released in response to mechanical stress to activate intracellular signaling pathways via interaction with cognate receptors [26,27]. Fragments of ECM constituents called matrikines, which may be generated by breakdown or proteolytic digestion, also have been reported to possess signaling properties that alter downstream cellular responses in a variety of tissues and disease settings [28,29,30]. ECM composition and remodeling also impact cell movement, such as during the metastasis of cancer cells [31,32]. Of greater relevance to the heart, the ECM impacts the incursion of circulatory cells such as macrophages during inflammation or after acute tissue damage [21,33]. Recent studies have demonstrated direct physical and/or paracrine interactions between cardiac fibroblasts and myocytes that likely have functional consequences in vivo [34,35,36,37,38]. However, the greatest impact of fibroblast activity in the heart is likely via their production, maintenance and degradation of ECM, and in times of stress or damage, their conversion to myofibroblasts.

## 3. Fibroblast to Myofibroblast Phenotype Conversion

In response to various indicators of cardiac stress or damage, including physical stretch, inflammatory mediators, growth factors and cytokines, cardiac fibroblasts may become activated, eventually undergoing a phenotype conversion to myofibroblasts [20,39]. Myofibroblasts have typically been defined by key phenotypic features including the *de novo* expression of markers including α-smooth muscle actin and periostin, increased production of ECM, and the ability to contract [17]. However, it is cell contractility and the production of stress fibers that truly defines the “myo”fibroblast phenotype, as not all cells that secrete increased ECM levels are necessarily myofibroblasts, although increased ECM production appears to be a general hallmark of these cells for the most part [40]. ECM over-production, i.e., fibrosis, occurs in many cardiac disease entities, including in response to pressure overload, after myocardial infarction, or as a long-term outcome of diabetes.

The conversion of fibroblasts to myofibroblasts was originally envisioned as a relatively simple process—cells were either fibroblasts, or they were myofibroblasts [41]. The concept of the proto-myofibroblast was introduced as an intermediate phenotype; these cells gained migratory capacity by increasing stress fiber formation to facilitate contraction, something not observed in non-activated fibroblasts [42]. Notably, these proto-myofibroblasts did not express α-smooth muscle actin. More recently, it has been recognized that the transition from fibroblasts to myofibroblasts is even more complicated. For example, during the activation of cardiac fibroblasts after myocardial infarction, in addition to the initial increase in migration (which precedes a subsequent decrease in migratory capability), these cells go through first a pro-inflammatory phenotype, followed by an anti-inflammatory phenotype [2]. Eventually, myofibroblasts arise, but even this phenotype is not necessarily the final end-point, as these cells may undergo a subsequent senescence, exiting the cell cycle but persisting at the site of injury [10]. It has been reported that myofibroblasts can be found in the infarct region many years after the infarction itself [43]. While a number of cells have been proposed as the source of myofibroblasts in the heart, including endothelial or epithelial cells and circulating progenitors, recent work has demonstrated that the vast majority of myofibroblasts arise from the phenotype conversion of resident fibroblasts [44]. Fibro-adipogenic progenitor cells have been reported in various tissues including skeletal muscle, and may represent another source of ECM producing cells after tissue damage or stress, although their potential role in the heart remains unclear [45].

While the signaling mechanisms governing fibroblast to myofibroblast conversion are not yet fully elucidated, much has been discovered to date. The TGFβ-Smad signaling pathway has long been known to be involved in this process, and is arguably one of the most potent inductive mechanisms: TGFβ drives fibroblast activation via the activation of phosphorylation of Smad2 and/or Smad3, which complex with Smad4, translocate to the nucleus and form a transcriptional complex that can directly bind to and transactivate key ECM genes such as those encoding type I collagen [20]. TGFβ may also work via a parallel non-canonical signaling pathway involving the activation of protein kinases such as p42/p44 MAPK [46]. A variety of growth factors have been implicated in inducing fibrosis in the heart and other tissues, including Connective Tissue Growth Factor CTGF/CCN2, angiotensin II and Platelet-Derived Growth Factor [20,47,48]. Mechanical stretch of fibroblasts also triggers their conversion to myofibroblasts, which is mimicked by the isolation and culture of primary fibroblasts on firm rather than soft tissue culture matrices [49,50,51,52,53]. Stretch-induced activation has been reported to involve Myocardin-Related Transcription Factor A and Serum Response Factor as downstream effectors [54]. Other intracellular signaling mediators of fibroblast to myofibroblast conversion include the Ski-Zeb2-Meox2 pathway, calcineurin/NFAT and p38α [55,56,57].

Over the past decade, our laboratory has demonstrated a sufficient and required role for the transcription factor scleraxis in fibroblast activation and the production of the cardiac ECM. Scleraxis is expressed in cardiac fibroblasts, its expression is increased in myofibroblasts, and it is highly expressed in the cardiac infarct scar compared to distal non-infarcted tissue [58]. Expression of scleraxis is induced by TGFβ and by Smad3 over-expression, and in turn, scleraxis binds to and directly transactivates the promoters of numerous ECM and fibrosis-related genes, including collagen Iα2, fibronectin, α-smooth muscle actin and MMP2 [17,58,59,60,61]. The critical importance of scleraxis in cardiac ECM production was revealed by analysis of the scleraxis-null murine myocardium: these hearts lacked 30–50% of the normal complement of ECM, and exhibited a ~50% decrease in fibroblast number [17]. The remaining fibroblasts, when isolated, had a greatly decreased capacity for ECM production, which could be rescued by scleraxis over-expression. The loss of fibroblasts may represent a defect in their production via epithelial-to-mesenchymal transition (EMT) of cardiac fibroblast precursors in the developing heart. We reported that scleraxis directly transactivates the genes encoding the potent EMT factors Snai1 and Twist1, that scleraxis alone can induce EMT of A549 epithelial cells, and that scleraxis was required for the ability of TGFβ to induce Snai1 and Twist1 expression [17,62].

Intriguingly, scleraxis seems to be required for many if not most of the functions of the TGFβ pro-fibrotic pathway in cardiac fibroblasts. In the absence of scleraxis, TGFβ appears to be incapable of inducing fibroblasts to produce ECM or to contract—essentially, scleraxis is required for TGFβ to induce fibroblast to myofibroblast transition [17]. Similarly, while cell stretch has been well-documented to induce fibroblast activation, scleraxis knockdown prevents stretch-induced changes in cardiac fibroblasts including hypertrophy, collagen production and α-smooth muscle actin expression [63]. We found that cell stretch induced scleraxis expression, which agrees with an earlier report that scleraxis expression correlates with mechanical force in tendons [64]. We hypothesized that scleraxis exerts its impact on the TGFβ pathway by acting as a required partner for Smad3. We have shown that scleraxis directly binds to Smad3 in cardiac fibroblasts, similar to a previous report that Smad3 forms a complex with scleraxis in tendon lysates [17,65]. We produced a scleraxis mutant lacking a DNA binding domain which acts in a dominant negative fashion to completely attenuate TGFβ-mediated collagen expression [58,59]. This scleraxis mutant may thus work by binding to and sequestering Smad3; the mutant is unable to bind to DNA, and thus may destabilize transcriptional complexes. Evidence for this is that increasing the amount of scleraxis mutant expressed in cardiac fibroblasts causes a dose-dependent decrease in the binding of both Smad3 and RNA polymerase II to the collagen Iα2 gene promoter [17]. Scleraxis is also constitutively phosphorylated on two conserved serine residues, and mutation of these sites to non-phosphorylatable moieties completely attenuates the ability of scleraxis to induce collagen gene expression by inhibiting scleraxis binding to target gene promoters [66].

Wound healing and fibrosis are intricately linked. Indeed, fibrosis can and does result when wound healing programs fail to execute or terminate correctly. Numerous lines of evidence suggest that scleraxis is involved in wound healing in various non-cardiac tissues. Several studies have demonstrated a required role for scleraxis in the healing of tendons, and it has been shown that treatment of damaged tendons with mesenchymal stem cells over-expressing scleraxis results in faster healing and stronger tendons than with conventional stem cell treatment [67,68,69,70]. Scleraxis expression increases in healing bone fractures, and scleraxis null mice exhibit a significant impairment in callus formation resulting in defective healing of fractures [71]. Conversely, elevated scleraxis expression has been noted in collagen-rich dermal keloids, and has been detected at elevated levels in the serum of patients with systemic sclerosis or idiopathic pulmonary fibrosis compared to healthy controls [72,73]. It is tempting to speculate, therefore, that scleraxis represents a key effector of wound healing pathways in multiple tissues, controlling the activation of fibroblasts to facilitate healing, and that its potential role in fibrosis stems from the excessive or prolonged activation of these pathways. Given its central role in the phenotype conversion of fibroblasts to myofibroblasts, scleraxis may represent a therapeutic target for fibrosis [74].

## 4. A Therapeutic Opportunity

The dramatic uptick in ECM production by myofibroblasts represents a metabolically expensive undertaking for the cell: large ECM proteins such as collagen have to be produced in high quantities, exported from the cell, and processed by intracellularly-produced but extracellularly active enzymes to produce the mature protein isoform. Each of these events requires a significant investment of cellular ATP, as does the increase in synthesis of myofibroblast contractile elements, stress fibers and markers. Given this requirement for increased cell metabolism, it is tempting to speculate that interference with these processes may provide a means to attenuate ECM production, to block fibroblast-to-myofibroblast phenotype conversion, or both—an intriguing opportunity for the development of anti-fibrotic medications.

In the heart, the metabolic processes involved in ECM synthesis and export remain poorly defined, but more is understood of these processes in the liver. Hepatic stellate cells that convert to myofibroblasts appear to derive their energy requirements from the catabolism of glutamine, the most abundant amino acid found in plasma, to α-ketoglutarate [75,76]. This process is analogous to an important energy metabolism pathway in cancer cells, and glutaminolysis inhibitors are being actively trialed as anti-cancer interventions [77]. Increased extracellular glutamine stimulates collagen synthesis [78,79]. Conversely, glutaminolysis inhibitors have been reported to block the conversion of hepatic stellate cells to myofibroblasts [80]. Importantly, blockade of glutamine breakdown showed promising effects on reducing liver fibrosis in an animal model [80].

It remains to be seen whether a similar approach will be successful in cardiac fibrosis, as the role of glutaminolysis in cardiac fibroblast to myofibroblast phenotype conversion has yet to be investigated. However, the overall strategy of interfering with fibroblast activation appears to be promising for slowing, stopping or perhaps even reversing fibrosis in the heart or other organs. Deletion of Smad3 attenuated cardiac fibrosis in a mouse model of hypertension [81]. Smad3 knockout also resulted in improved cutaneous wound healing, with evidence of reduced local inflammation [82]. Targeting fibroblast activation—by targeting pro-fibrotic transcription factors like Smad3 or scleraxis, or by targeting energy metabolism as with glutaminolysis inhibitors—represents an exciting new frontier for anti-fibrotic development.

As with any new approach, there are challenges. The activation of fibroblasts to become myofibroblasts is an important aspect of wound healing, and a recent report suggests that loss of Smad3 specifically in cardiac myofibroblasts results in reduced cardiac systolic function and accelerated matrix degradation [83]. Anti-fibrotics may thus exert adverse effects on salutary processes, although it may be possible to address this issue by interrupting anti-fibrotic delivery, developing tissue-specific approaches, or targeting specific signaling pathways. This may be a particular concern in the setting of myocardial infarction, as it would be critical to deliver anti-fibrotics well after the infarct scar has formed and matured to prevent aneurysm. Given the tremendous heterogeneity of fibroblasts across tissues and organs, there is also the possibility that the fibroblast-to-myofibroblast transition process varies widely as well, with different regulators or effectors of the process. This concern is less easily dispelled, although it is noteworthy that the TGFβ/Smad pathway appears to be pro-fibrotic in many different tissue types, and that scleraxis has been reported to be highly expressed in collagen-rich dermal keloids and muscle tendons, suggesting some degree of conservation of action across cell and tissue types [72,84]. Ultimately, there is a tremendous opportunity to make significant inroads in the production of anti-fibrotic medications, and even the possibility that by targeting the correct pathway, a single medication may be effective for treating fibrosis in many different organs. Attention is urgently needed for this clinical problem that remains largely devoid of treatment options.

## Abbreviations

ECM, extracellular matrix; EMT, epithelial-to-mesenchymal transition; MMP, matrix metalloproteinase; TGFβ, Transforming Growth Factor β
